# Total Body Irradiation (TBI) using Helical Tomotherapy in children and young adults undergoing stem cell transplantation

**DOI:** 10.1186/1748-717X-8-92

**Published:** 2013-04-15

**Authors:** Arne Gruen, Wolfram Ebell, Waldemar Wlodarczyk, Oliver Neumann, Joern Sven Kuehl, Carmen Stromberger, Volker Budach, Simone Marnitz

**Affiliations:** 1 Department of Radiation Oncology, Charité – University Medicine Berlin, Augustenburger Platz 1, 13353 Berlin, Germany; 2 Department of Pediatric Oncology, Charité – University Medicine Berlin, Augustenburger Platz 1, 13353 Berlin, Germany

## Abstract

**Background:**

Establishing Total Body Irradiation (TBI) using Helical Tomotherapy (HT) to gain better control over dose distribution and homogeneity and to individually spare organs at risk. Because of their limited body length the technique seems especially eligible in juvenile patients.

**Patients and methods:**

The cohort consisted of 10 patients, 6 female and 4 male, aged 4 - 22 y with acute lymphoblastic- (ALL) or acute myeloic leukemia (AML). All patients presented with high risk disease features. Body length in treatment position ranged from 110–180 cm. Two Gy single dose was applied BID to a total dose of 12 Gy. Dose volume constraint for the PTV was 95% dose coverage for 95% of the volume. The lungs were spared to a mean dose of [less than or equal to] 10 Gy. Patients were positioned in a vac-loc bag in supine position with a 3-point head mask.

**Results:**

Average D95 to the PTV was 11.7 Gy corresponding to a mean coverage of the PTV of 97.5%. Dmean for the lungs was 9.14 Gy. Grade 3–4 side effects were not observed.

**Conclusions:**

TBI using HT is feasible and well tolerated. A benefit could be demonstrated with regard to dose distribution and homogeneity and the selective dose-reduction to organs at risk.

## Introduction

Among the downsides of classic TBI are the long application time, non-conformality of beam-application with the inability to individually spare organs at risk (OARs) and hence – its` acute and late toxicity [1-5]. With the development and clinical use of highly conformal radiation technologies such as Helical Tomotherapy™ [6-8] different working groups have investigated their role and feasibility in TBI, total marrow- (TMI) or total lymphoid irradiation (TLI) [9-14]. It could be shown that TBI using HT is feasible and offers advantages over the standard LINAC-based approach. Individual RT-planning allows for better control over and improvement of dose-distribution on target-structures and OARs. With the helical beam-delivery one can increase both conformality and homogeneity in target-dose distribution. MV-CT-based guidance provides image-adapted beam-delivery after correction for patient movement and set-up errors.

**Figure 1:**
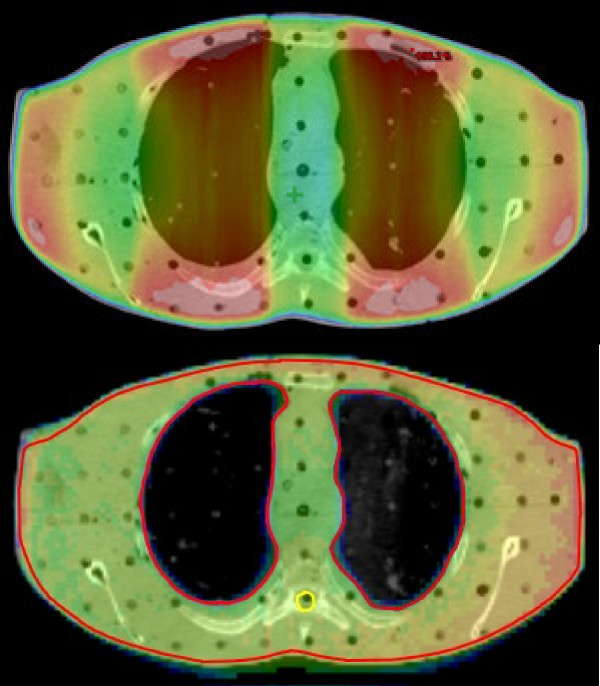
Comparison of the dose distribution over the lungs in an Alderson phantom using either a Linac with lung attenuators (upper part) or Helical Tomotherapy (lower part).

The goal of this study was the establishment of HT-based TBI at our institute. As of now our standard procedure for TBI was Linac-based using a translation bunk and lung attenuators for lung shielding. Using HT for TBI we strived for individual control over dose-distribution for optimal target coverage and highly conformal sparing of organs at risk such as the lungs at the same time to possibly decrease acute as well as late treatment sequelae (Figure 1). By shortening overall treatment time patients` comfort should be enhanced. Because of their limited body length children and young adults seemed especially eligible for the technique. To guarantee comparability we adapted dose fractionation and dose planning-constraints from our standard LINAC-based TBI-protocol.

## Methods

### Consent

All patients were informed about the treatment and it`s possible adverse events and about necessary diagnostics prior to treatment. Written consent by either the patient himself or parents or legal guardians was obtained in all cases.

### Positioning/planning-CT

Patients were immobilised in supine position using customised thermoplastic 3-point masks (Orfit Efficast, Orfit Industries America, 350 Jericho Turnpike, Suite 101, Jericho, New York 11753) for head support and a vacuum cradle (Elekta BlueBAGTM, Henderson, NV, 89074) for body support on an adjustable combined board. Planning CT images (Siemens Somatom Sensation Open, Siemens Medical Solutions USA, Inc., Malvern, PA 19355–1406) were acquired in supine position with 5 mm slices. In case of patients no longer than 145 cm in body length, CT images were acquired head-first from the patients’ vertex to the toes. For localization purposes, fiducial markers (Beekley CT-SPOTS^®^ Crosshair, Beekley Corporation, Bristol CT 06010, USA) were attached in at least three axial planes: in the regions of the head, thorax/abdomen and knees. For patients exceeding 145 cm body length two CT data sets were acquired. One covered the range between the patients’ vertex and the lower thigh. In this case apart from the markers at head and thorax/abdomen, a third fiducial set was placed in the PTV cut plane, which was assigned to the mid of the upper thigh. The second CT scan covered the range between the patients’ toes and the upper thigh. Here the fiducials were placed in the knee plane and again in the cut plane. After acquisition of the CTs the position of the fiducial marker was transferred onto the patient using a felt pen. The marker itself is then removed. To prevent blurring of the skin marks a transparent foil was applied.

### Contouring

**Figure 2:**
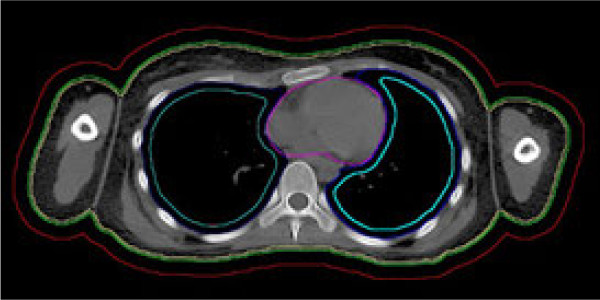
**Contours: body (green).** For the prevention of dose calculation artifacts the PTV1 equals the body contour with an internal margin of 2 mm (yellow). To prevent underdosing in the case of patient movement during treatment a second PTV (PTV2) with minor importance equals the body plus an external margin of 10 mm (red). Lung contours (dark blue). Depending on patient and organ size an internal margin has been created into the lungs to prevent underdosing on the ribs due to respiratory motion. Lung sparing constraints thus refer to the lungs minus the individual internal margin of 0.5-1 cm (light blue). Heart (purple).

For contouring and planning the Varian Eclipse treatment planning system (TPS), Version 8.8 (Varian Medical Systems, Inc., Palo Alto, CA 94304–1038, USA) was used. To avoid dose calculation artifacts due to proximity of the body surface we created an inner margin of 2 mm beneath the outer surface of the body as PTV1 for TBI. To avoid underdosage of the ribs and the thoracic wall due to respiratory motion we created an additional inner margin of 5–10 mm (depending on patient and thus on organ size) into the lungs. This resulted in a volume reduction of the lungs as an OAR (Figure 2). Following lung dose constraints refer to the lung subtracted by the individual internal margin (IM). In case of split treatments the PTV1 ended 2 cm set back from the actual cut plane in both the upper body and lower body plan to ensure a homogeneous dose transition between the upper-and lower body plans as determined previously in experimental studies on a cheese phantom. (Data will be presented elsewhere). To reduce the susceptibility of delivered dose distributions to setup imperfections and patient motion a virtual air bolus of 10 mm thickness was added to the patient surface as an additional target volume (PTV2) with the prescribed dose of 12 Gy but lower importance (Figure 2).

### Dose prescription and fractionation

Dose prescription to the PTV was 2 Gy single doses delivered twice a day (BID) with an interfraction interval of at least eight hours on three consecutive days to a total dose of 12 Gy. Constraints to be fulfilled were the coverage of 95% of the PTV1 by 95% of the prescribed dose (12 Gy) and the suppression of the lung dose to a mean dose (Dmean) of no more than 10 Gy and a minimum dose (Dmin) of 8 Gy [15]. Planning criteria were the homogenous coverage of the PTV by the prescribed dose and dose to the lungs. Dose peaks (hot spots) were tolerated only if they were located in the bone marrow or musculature. 3-D data sets with contours were transferred to the Hi-Art Treatment Planning System (TPS) (Tomotherapy Inc., Madison, WI 53717–1954 USA) for planning. Here we could benefit from previous parametric studies and apply the heuristic rules for planning parameters (especially for Pitch (PI)) gained there without carrying out extensive calculations every time (Table 1).

**Table 1:** Planning parameters

**Pt. no.**	**Single dose**	**Fractions**	**Total dose**	**Lung sparing y/n**	**Pitch**	**Pitch legs**	**MF**	**MF legs**	**Field width**	**t**
**1**	2	6	12	y	0.43	/	1.5	/	5	1236 s (=20.6 min)
**2**	2	6	12	y	0.43	/	1.3	/	5	815 s (=13.6 min)
**3**	2	6	12	y	0.43	0.43	2.1	1.1	5	1812 s (=30.2 min)
**4**	2	6	12	y	0.3	0.43	2	1.3	5	1791 s (=29.9 min)
**5**	2	6	12	y	0.3	0.43	2	1.1	5	1851 s (=30.9 min)
**6**	2	6	12	y	0.45	/	1.22	/	5	916 s (=15.3 min)
**7**	2	6	12	y	0.35	0.4	2	1.1	5	1738 s (=29.0 min)
**8**	2	6	12	y	0.3	0.4	2	1.1	5	1723 s (=28.7 min)
**9**	2	6	12	y	0.3	0.4	2	1.1	5	1649 s (=27.5 min)
**10**	2	6	12	y	0.4	0.4	2	2	5	2374 s (=39.6 min)

All patients were treated with single doses of 2 Gy applied twice a day with an interfraction interval of at least eight hours on three consecutive days to a total dose of 12 Gy with lung sparing to a Dmean of 10 Gy and a Dmin of 8 Gy. Pitch values of 0.3-0.45 and modulation factors (MF) of 1.1-2.1 were used. Field width of 5 cm.

As back-up we generated conventional LINAC-based TBI plans for all patients. For this purpose additional CT data-sets in supine and prone position were acquired. These data-sets were used for TBI planning using templates and resulted in specific values for translation speed of the bunk and thickness of individual lung attenuators to suppress the lung dose to a Dmean of approximately 10 Gy.

### Treatment

Since we opted for a one-machine solution to avoid transfer of the patient to another machine during treatment we divided the PTV of patients with body length exceeding 145 cm into two parts and delivered the TBI in two successive sessions: (i) head first from vertex to the cut plane and (ii) after repositioning: feet first from toes to the cut plane. For all other patients each TBI fraction was delivered in one session. To guarantee dose build-up on bony structures lying within close proximity to the skin we put 1 cm water equivalent flab-material on the hands, sternum and clavicles of the patients.

### Quality assurance

**Figure 3:**
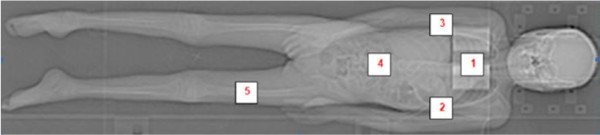
Standard localization of TLDs: Sternum (1); Axilla right (2); Axilla left (3); Abdomen (4); Upper thigh right (5).

For the purpose of quality assurance (QA), two different procedures of absolute dosimetry were always applied. On the one hand measurements with an ionization chamber in the “cheese” phantom and concomitant measurements with thermo- luminescent detectors (TLDs) at different reference points on the patients’ body (Figure 3 and Table 2).

**Table 2:** TLD measurement results

**Patient no.**	**Sternum**	**Axilla right**	**Axilla left**	**Abdomen**	**Upper thigh right**
**1**	1.95	2.09	/	1.81	/
**2**	/	2.18	2.13	1.91	2.01
**3**	2.06	2.03	1.9	2.05	3.29
**4**	2.05	1.97	1.93	/	/
**5**	1.99	2.03	1.88	2.05	2.33
**6**	/	2.04	2.03	1.94	2.1
**7**	1.92	1.87	1.96	1.97	2.86
**8**	2.2	1.89	2.38	2.36	2.29
**9**	2.29	2.29	2.86	2.28	2.59
**10**	2.10	2.05	2.04	1.99	3.14

Exemplary data taken from the 1st fraction of each patient. No TLDs used or TLD-dislocation in some cases without proper values (/). “Upper thigh right” position equals the cut plane where upper and lower body PTVs meet and supposed to merge homogenously, 3.29 Gy in patient number 3 and 3.14 Gy in patient number 10 due to merge of upper and lower plan and consecutive overdosing in cut plane which was accepted to avoid under-dosing.

### Positioning/image guidance

Positioning was verified prior to treatment using megavoltage (MV)-CTs of either the pre-defined cranio-thoracal or the pelvic area [16]. Additional MV-CTs of the knee area were needed in lower body plans in patients receiving a split-plan treatment. Set up errors were corrected for immediately.

### Anesthesia/monitoring

Since for many of the patients the stress level during the procedure was fairly high due to the strict immobilization and the long treatment times most of our patients needed either sedation or anesthesia (using benzodiazepines, propofol or ketamines in individual dosages). One patient needed general intubation anesthesia. Sedation and anesthesia were applied and overseen by pediatric oncologists and anesthetists. Vital parameters (heart rate, oxygen saturation) during treatment could be monitored using a pulse oximeter (NellcorTM OximaxTM N-65TM, Tyco Healthcare Group LP, Nellcor Puritan Bennett Group, Pleasanton, CA, USA) and the in-room camera and microphone system (EE811A, Schneider Intercom GmbH, Erkrath, Germany).

## Patients

Ten patients underwent TBI treatment using HT. Patients´ characteristics and individual risk factors are shown in Table 3. All patients presented with high-risk features such as genetic predictors for high risk of recurrence such as bcr-abl positivity or non-response to chemotherapy. Five patients had recurrent disease, one of them presented with a second recurrence. Age of the patients ranged from 4 – 22 years with an average of 12.9 years of age. The cohort consisted of 6 female and 4 male patients. Body length in treatment position (supine position, feet outstretched) ranged from 110 – 180 cm. Apart from mild chemotherapy induced toxicity such as fatigue, loss of appetite or alopecia neither of the patients presented with excess morbidity going into treatment.

**Table 3:** Patients` characteristics

**Patient no.**	**Age at treatment (years)**	**Diagnosis**	**Additional risk-factors**	**Prior radiation treatment**	**Body length in treatment position (cm)**
**1**	8	ALL	BCR-ABL^+^	/	154
**2**	4	ALL	BCR-ABL^+^	/	110
**3**	18	ALL	Non-response day 28 of induction therapy	/	180
**4**	22	AML in CML	M-BCR-ABL^+^	/	180
**5**	18	ALL Recurrence	/	/	172
**6**	7	ALL Recurrence	Biphenotypic leukemia	/	130
**7**	13	ALL 2nd Recurrence	/	12 Gy Whole Brain; 24 Gy Scrotum	164
**8**	14	ALL Recurrence	/	/	172
**9**	9	ALL Recurrence	/	12 Gy Whole Brain	158
**10**	12	ALL Recurrence	/	18 Gy Whole Brain	152

Patient age ranged from 4–22 years. Most of the patients presented with ALL (acute lymphoblastic leukemia) in first or second remission, one patient presented with an acute blast crisis (AML: acute myeloic leukemia) in a CML (chronic myeloic leukemia) Risk factors such as positivity for the bcr-abl gene fusion product were seen in some case, two patients had had prior radiation therapy. Body length in treatment position (supine, feet outstretched) ranged from 110–180 cm.

## Results

**Figure 4:**
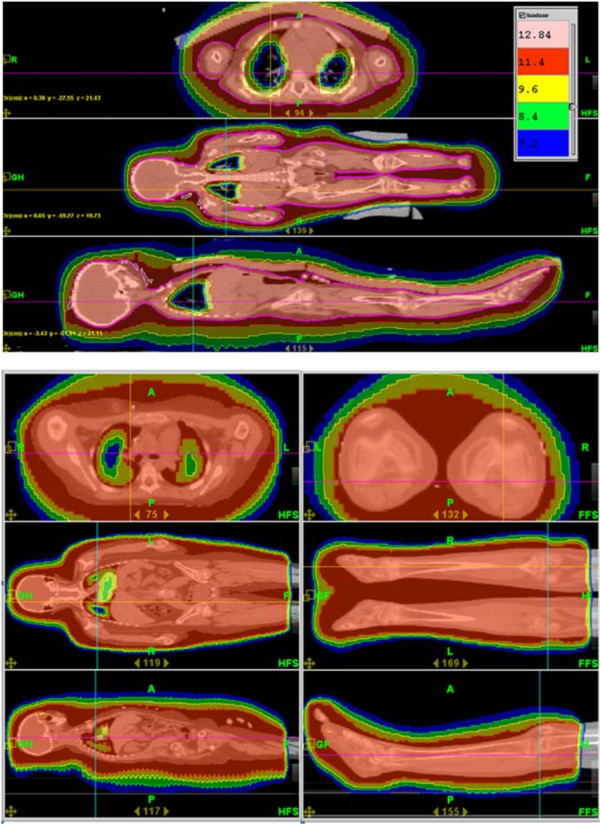
**Dose distribution.** (**a**) transverse, coronal and sagittal dose planes of a non-split TBI-plan (**b**) transverse, coronal and sagittal dose planes of the upper and lower part of a split TBI-plan.

The average dose covering 95% of the target (D95) to the PTV1 in all ten patients was 11.7 Gy corresponding to a mean coverage of the PTV by 97.5% (Figure 4a and b). Actual average Dmean for the lungs was 9.27 Gy and 9.25 Gy for left and right lung respectively (Table 4). Correlation between actual measured doses by TLD and calculated doses was satisfactory with no under-dosing observed (Tables 2 and 4). Dose peaks of up to 130% of the prescribed dose were seen in small volumes. All treatment plans were checked for dose peaks exceeding the prescribed dose by more than 107%. Dose peaks were accepted if only small volumes in non-critical target structures were affected. Beam-on time ranged from 13.6 min to 20.6 min, average: 17.1 min for the non-split plans vs. 28.7 min to 39.6 min (average: 34.2 min) for the split plans. Due to their body length in treatment position (supine, feet outstretched) of ≤ 145 cm three patients could be treated with a non-split TBI plan as compared to seven patients for whom the procedure had to be split into an upper body- and lower body plan connecting in the upper thigh area.

**Table 4:** DVH parameters

**Patient no.**	**PTV1 D95 (Gy)**	**PTV1 lower body D95 (Gy)**	**PTV1 D mean (Gy)**	**PTV1 lower body Dmean (Gy)**	**PTV1 D5 (Gy)**	**PTV1 lower body D5 (Gy)**	**Lung L - IM Dmean (Gy)**	**Lung R - IM Dmean (Gy)**
**1**	11.70	/	11.92	/	12.30	/	7.72	7.55
**2**	11.80	/	12.02	/	12.30	/	8.19	8.00
**3**	11.70	11.60	12.11	12.11	12.40	12.70	9.14	9.04
**4**	11.80	11.80	11.93	12.02	12.30	12.20	9.01	9.06
**5**	11.80	11.80	11.98	12.10	12.30	12.70	9.18	8.94
**6**	11.60	/	12.06	/	12.70	/	9.63	9.82
**7**	11.60	11.50	12.10	12.08	12.80	13.30	10.36	10.06
**8**	11.70	11.70	12.08	12.17	12.60	12.90	9.98	10.12
**9**	11.80	11.70	12.20	12.14	13.50	13.00	9.71	9.72
**10**	11.70	11.80	12.00	12.00	12.80	13.00	9.80	10.20
**Range**	11.60-11.80	11.5-11.8	11.92-12.20	12.02-12.17	12.30-13.50	12.20-13.30	7.72-10.36	7.55-10.12
**Average**	**11.72**	**11.70**	**12.04**	**12.09**	**12.60**	**12.83**	**9.27**	**9.25**

Actual overall “in-room”-time (patient going into the treatment room to patient leaving) regularly exceeded actual beam-on considerably. The pronounced differences between net beam-on time and actual overall treatment time were due to the positioning procedures including MV-CTs, correction for set-up errors and the sometimes elaborate anesthetic measures and finally due to treatment interruptions caused by machine-failure or down time. Three out of 54 overall fractions had to be applied LINAC-based due to HT down time. The largest share of hardware-associated interruptions stemmed from MLC-errors that could be overwritten without compromising the treatment. In two patients the treatment had to be prolonged into a fourth day to complete the treatment.

Most of our patients complained of fatigue, loss of appetite or nausea even before the onset of therapy due to prior cytotoxic treatment. These symptoms were only slightly aggravated by TBI. Overall acute morbidity of TBI was low corresponding to only mild Grade 1 – 2 side effects in all 10 patients, grade 3–4 side effects were not observed (Table 5).

**Table 5:** Acute toxicity

**Pt. no.**	**Grade 1–2 toxicity**	**Grade 3-4**
**1**	Mucositis, Fatigue	/
**2**	Fatigue	/
**3**	Loss of appetite, Xerostomia, Diarrhea, Fatigue, Erythema, Conjunctivitis	/
**4**	Nausea	/
**5**	Xerostomie, Parotitis, Headache, Fatigue	/
**6**	Loss of appetite, Fatigue, Nausea, Dysgeusia	/
**7**	Loss of appetite, Nausea, Headache, Neck pain	/
**8**	Loss of appetite, Nausea, Fatigue	/
**9**	Xerostomia	/

Overall acute toxicity was low with only grade 1–2 morbidity. Grade 3–4 toxicity was not observed. Toxicity scoring by CTCAE v4.0.

With 1 to 15 months of follow up eight of ten patients are in stable remission without further radiation-related morbidity. One patient died immediately after transplantation due to bacterial sepsis and consecutive multi-organ failure and a second patient died three month after transplantation due to uncontrollable graft versus host disease (GVHD) grade IV.

## Discussion

In the past decades cure rates in paediatric oncology have been increased. Especially in those cohorts with high cure rates and long-term survivors all efforts should be made with regard to the reduction of acute and chronic therapy related morbidity. Most authors report on LINAC-based procedures with a simple technique using ap/pa portals, although other attempts at treating the patient over the whole axis using HT, as we did, have been published [17,18]. The concept of HT for TBI offers advantages over conventional LINAC-based TBI with higher conformality due to the 360° of beam application versus the standard fixed beam approach (Figure 1). Further it allows for individual sparing of organs at high risk of radiation induced toxicity such as the lungs. Since the main goal of this study was to establish HT in TBI at our institute we used the same fractionation and dose constraints as in our standard LINAC-based TBI protocol only adding a minimum dose for the lungs to prevent underdosing and thus a possible increase in disease relapses. Highly conformal lung sparing could be achieved with mean lung doses of no more than 10 Gy. Overall morbidity during treatment was low corresponding to only mild grade 1–2 side effects which is in line with the results seen by other groups such as Schultheiss et al. [11], grade 3 – 4 side effects were not observed [14]. No lung toxicity was observed and is still not reported on with up to 15 months of follow-up.

A disadvantage of HT is the limited translation length of the table, allowing irradiable PTV lengths of approximately 145 cm. All patients exceeding 145 cm body length need a solution concerning the irradiation technique for the lower part of the body. The interruption of treatment and shift of the patient from one machine to another implies discomfort for the patient and is a potential source of QA-problems. That is why we decided to use a “single-machine”-solution and devised separate lower body plans for patients with exceeding body length. Other groups kept the legs of patients with exceeding body length in a folded position in a vac-loc bag [9], others are using ap/pa portals of a Linac for TBI and are applying Helical Tomotherapy Total Marrow Irradiation only as a boost [18].

We could show that TBI using HT is an interdisciplinary challenge, but feasible as a one-machine-solution even for patients with body length exceeding 145 cm.

Planning constraints derived from conventional LINAC-based TBI could be fulfilled. We opted for a minimal lung dose of 8 Gy to prevent underdosing and thus increasing possible relapse rates [19]. We verified calculated doses by TLD-measurements on defined localizations during treatment on the patients. Measured doses generally corresponded well to the calculated doses. No under-dosing was observed. Dose maxima within the PTV were acceptable. Set-up errors or patient movement during treatment of up to 1 cm were anticipated by using a double PTV concept and creating a second PTV (PTV2) of 10 mm around the patient, thereby creating a safety margin where dose would be deposited in case the patient moved.

A reduction of overall treatment time compared to our standard LINAC-based TBI approach could not be achieved. Necessary patient preparation with image guidance and correction for set-up errors, sedation or anesthesia where necessary added to the complexity of the procedure and called for increased man-power with not only radiation oncologists, physicists and radiographers but sometimes also paediatric oncologists, anesthesists and their personnel overseeing the treatment. The elevated stress level for the patients caused by the strict immobilization, the claustrophobic interior of the bore and the loud machine noise made up some of the draw-backs of the procedure.

Technical difficulties, this mainly being MLC-errors, further prolonged overall treatment time. Excess maintenance could only partly compensate for the problem thus clearly leaving room for improvement in technical reliability.

## Conclusion

It could be demonstrated that HT allows for excellent target coverage of the PTV with individual sparing of organs at risk such as the lungs. Only mild side effects were observed. Longer treatment times and technical difficulties leave room for further improvements of the technique. Nevertheless, HT seems to be a promising tool for optimizing radiation therapy in pediatric oncology.

## Competing interests

The authors declare that they have no competing interests.

## Authors’ contributions

AG conceived and developed the project, informed and obtained written consent of the patients or their guardians, did the contouring, supervised RT application, drafted the manuscript. WE developed and supervised the project on the pediatric oncology side. WW developed the RT algorhythms, did the RT-planning. ON did the RT-planning. JSK developed and supervised the project on the pediatric oncology side, supervised RT application. CS did the contouring, supervised RT application. VB conceived and developed the projectSM conceived and developed the project, edited the manuscript. All authors read and approved the final manuscript.
